# Supplementary material for: Global Transcriptome Analyses of Cellular and Viral mRNAs during HAdV-C5 Infection Highlight New Aspects of Viral mRNA Biogenesis and Cytoplasmic Viral mRNA Accumulations
Source: Viruses. 2022 Nov 1;14(11):2428. doi: 10.3390/v14112428 (PMC9692883; doi:10.3390/v14112428)
Supplement: Supplementary file 1 [file viruses-14-02428-s001.zip › viruses-1955083-supplementary.pdf]

viruses

MDPI

Supplementary Information

Global Transcriptome Analyses of Cellular and Viral mRNAs During HAdV-C5 Infection Highlight New Aspects of Viral mRNA Biogenesis and Cytoplasmic Viral mRNA Accumulations

Margarita Valdés Alemán, Luca D. Bertzbach, Thomas Speiseder, Wing Hang Ip, Ramón A. González, and Thomas Dobner

Table S1. HAdV-C5 transcriptome annotation.

Transcript	Region (nt positions)	
E1A 10S	499..637, 854..974, 1229..1545	a
E1A 11S	499..637, 854..1112, 1229..1545	a
E1A 12S	499..974, 1229..1545	
E1A 13S	499..1112, 1229..1545	
E1A 9S	499..637, 1229..1545	a
E1B new	1702..2255, 3598..4070	a
E1B-13S	1702..2255, 3595..4070	
E1B-14.5S	1702..2255, 3218..3510, 3595..4070	
E1B-14S	1702..2255, 3276..3510, 3595..4070	
E1B-179R	1702..2324, 3276..3510, 3595..4070	
E1B-22S	1702..3510, 3595..4070	
E2A DBP	22443..24032 (rc)	
E2A	22374..24032, 24667..24745, 26977..27045 (rc)	a
E2A new1	22374..24042, 24146..24745, 26977..27045 (rc)	a
E2A new2	22374..24331, 24667..24745, 26977..27045 (rc)	a
E2A-L	22374..24032, 24667..24745, 25838..25909 (rc)	a
E2B (Ad pol)	4060..8785, 14111..14299, 24667..24745, 26977..27045 (rc)	a
E2B 120K DNA polymerase	5197..8784, 14112..14120 (rc)	
E2B IVa2	4091..5427, 5706..5718 (rc)	
E2B new	4060..20695, 20823..24745, 26977..27045 (rc)	a
E2B pTP (75K)	4060..10590, 14111..14299, 26977..27045 (rc)	a
E2B pTP 75K	8583..10589, 14112..14120 (rc)	
E3 12.5K	27852..28175	
E3 14.7K (truncated form, 5' 24 nts deleted)	28593..28955	
E3 CR1-alpha0	28541..28592	
E3-1	27562..28592	
E4orf1_1 late	30932..31308, 32020..33642, 33658..33724 (rc)	b
E4orf1_2 late	30932..31308, 32020..32198, 32719..33642, 33658..33724 (rc)	b

E4orf2 early	30932..33208, 33658..33724 (rc)	b
E4orf3 early	30932..32848, 33658..33724 (rc)	b
E4orf3 late	30932..31308, 32020..32848, 33658..33724 (rc)	b
E4orf3/4 late	30932..31308, 32020..32198, 32719..32848, 33658..33724 (rc)	b
E4orf4 early	30932..32548, 33658..33724 (rc)	b
E4orf4 late	30932..31308, 32020..32548, 33658..33724 (rc)	b
E4orf6 new	30932..32358, 33658..33724 (rc)	a
E4orf6_1 early	30932..32445, 33658..33724 (rc)	b
E4orf6_2 late	30932..32198, 32404..32548, 33658..33724 (rc)	b
E4orf6/7_1 late	30932..31308, 32020..32445, 33658..33724 (rc)	b
E4orf6/7_2 late	30932..31308, 32020..32198, 33658..33724 (rc)	b
L1 13.6K	7978..8427	c
L1 52, 55K	6049..6090, 7110..7183, 9643..9734, 11050..12297	
L1 pIIIa	6049..6090, 7110..7183, 9643..9734, 12318..14075	
L2 pIII (penton base)	6049..6090, 7110..7183, 9643..9734, 14157..15872	
L2 pV	6049..6090, 7110..7183, 9643..9734, 16545..17651	
L2 pVII	6049..6090, 7110..7183, 9643..9734, 15879..16475	
L2 pVII mRNA1	6049..6090, 7110..7183, 9643..9734, 15659..16475	a
L2 pVII mRNA2	6049..6090, 7110..7183, 9643..9734, 15665..16475	a
L2 pVII mRNA3	6049..6090, 7110..7183, 9643..9734, 15729..16475	a
L2 pX (μ)	6049..6090, 7110..7183, 9643..9734, 17679..17921	
L3 23K protease	6049..6090, 7110..7183, 9643..9734, 21733..22347	
L3 hexon	6049..6090, 7110..7183, 9643..9734, 18842..21700	
L3 pVI	6049..6090, 7110..7183, 9643..9734, 18004..18756	
L4 100K	6049..6090, 7110..7183, 9643..9734, 24061..26484	
L4 22K	6049..6090, 7110..7183, 9643..9734, 26195..26779	
L4 33K	6049..6090, 7110..7183, 9643..9734, 26195..26510, 26713..27080	
L4 pVIII	6049..6090, 7110..7183, 9643..9734, 27168..27851	
L5 pIV fiber	6049..6090, 7110..7183, 9643..9734, 29158..30903	
L5 pIV fiber mRNA5	6049..6090, 7110..7183, 7951..8392, 9643..9734, 27800..27934, 29158..30903	a
L5 pIV fiber mRNA8	6049..6090, 7110..7183, 7951..8392, 9643..9734, 27800..27934, 29158..30903	a
pIX	3609..4031	
predicted E4orf5	30932..31308, 32031..32123, 33658..33724 (rc)	
UXP	22374..24042, 24667..24745, 28984..29147 (rc)	a

[1] nt, nucleotide; rc, reverse complement; a, predicted according to [1] and to Ad2 (GenBank accession number NC_001405); b, reported in [2]; c, i-leader, (GenBank accession number AFS50547.1).

Table S2. Cellular mRNAs clustered by cyto/nuc ratios. Listed are transcripts belonging to each of the clusters (A-G) illustrated in Figure 3.

A	B	C	D	E	F	G
AGA_2	AHNAK2_4	ATL2_13	AKR1B1_14	ACLY_2	ANXA1_3	ABCC3_9
AGR2_2	ANXA4_5	CD46_19	ASAH1_8	ARRB2_10	APP_2	ACTB_10
ARPC2_4	ANXA5_4	CLU_12	ATP6AP1_3	ELOVL5_6	ARGLU1_4	ADIPOR1_1
ASAH1_12	ATP1B3_11	F5_2	CALU_7	ETFA_5	ASAH1_9	ALDH1A1_2
ATP5I_5	BZW1_3	FN1_9	FADS2_3	HNRNPK_5	ATP1A1_8	ANXA1_7
AUP1_2	CALU_4	FTH1_4	FLNA_12	LAMB1_8	C1R_11	ANXA2_18
B2M_9	CANX_11	GJA1_1	NCL_3	MSMO1_2	CALR_1	ARL6IP4_11
BRI3_3	CAPN2_8	HECTD1_17	P4HB_16	TGFBI_14	CALR_5	ATP2A2_7
C1GALT1C1_2	CCT5_3	HSP90B1_2	RPS26_2		CCPG1_13	ATP5B_1
C5orf15_2	CCT8_9	NIT2_7	RPSA_9		CCT6A_10	ATP6AP2_7
CALU_8	CD44_17	PTGS2_1	SAT1_3		CLTC_4	ATRAID_7
CD59_3	CFH_4	RNF149_3	SDC1_2		CSE1L_4	BEST1_8
CLGN_3	CTSL_4	SDF2_6	TPM3_22		EIF4A2_6	C1S_19
CP_8	DDOST_5	SEP15_3	VCAN_10		HEXB_3	CA12_4
CTSC_4	DDOST_6	SPP1_9			HNRNPK_10	CCT6A_3
CTSS_1	ERP44_1				HSP90B1_3	CHMP3_9
CYP24A1_2	GNB2L1_32				HSP90B1_9	CLU_1
DNAJB9_1	HM13_24				IL18_5	CP_10
DNAJC2_4	IDH1_7				JAG1_1_3	CTD-2139B15.2_1
EPT1_1	ITFG1_9				MRPS5_1	CTD-2354A18.1_1
FAS_5	ITGB1_3				NRCAM_14	CTD-2354A18.1_3
FGB_5	MATR3_39				NRP1_5	CTDNEP1_12
FTH1_10	MFGE8_15				OGT_3	CTDNEP1_6
FTH1_2	NUCB2_17				PPIA_8	CTSF_3
FTH1_3	PON2_2				RPN2_8	CTSL_3
FTH1_5	POR_9				RPS24_8	CTSL_5
FTH1_6	PRKDC_9				SAT2_11	CXCL5_1
FTH1_7	PSAP_1				SLC16A1_1	DBNDD1_4
FTL_1	RDX_18				SLC25A3_10	DEGS1_1
GCNT3_10	RPL12_5				SLC2A1_4	DHRS7_7
GGH_2	RTN4_3				TM2D1_8	DKK1_2
GINM1_2	SERPINE1_2				TMBIM6_4	DMTN_2
GNAS_4	SERPINE2_7				VMP1_6	DSG2_1
GPS2_4	SLC27A2_2				VTI1B_5	EIF4EBP2_1
GSK3A_3	STT3A_8				XPO1_3	EIF4G2_28
HEXB_12	TFRC_2					EMC3_1
HNRNPDL_5	TGFBI_7					EPAS1_2
HNRNPUL1_13	TM9SF2_1					EPN1_4
HSD17B12_8	UBE2V1_20					EWSR1_17

HSPA8_9	VTN_2	F2RL1_2
HSPB1_2		FAM136A_4
IDS_1_6		FBL_1
IFNGR1_3		FLNB_1
LAMP2_2		FN1_16
LAMP2_4		FN1_20
LIPA_6		FTH1_1
LITAF_14		FTH1_8
MAGT1_2		GANAB_9
MGST2_3		GNAS_44
MTUS1_30		GPX8_7
NEU1_2		GUK1_31
NME4_6		HDLBP_31
NPTN_4		HEXA_9
OCIAD1_12		HLA-B_1
PABPC1_13		HMGB1_3
PON2_11		HNRNPA2B1_7
PRCP_3		HNRNPH1_36
PRDX4_2		HNRNPL_13
PTRH2_5		HRAS_7
RDH11_11		HSPA5_1
RP11-396K3.1_2		HTRA1_1
RPL36A_4		IDH1_9
RPL3_17		IGFBP3_8
RPS2_5		IRAK1_11
SAT1_8		ITGA3_6
SEC11A_7		ITGB1_1
SLC35F5_2		ITM2B_4
SLC38A2_6		JKAMP_11
SLC3A2_5		LAMB1_13
SRGN_2		LAMB2_11
SRPRB_5		LAPTM4A_2
SRSF7_2		LBR_8
SRSF7_6		LUC7L3_10
STK24_7		MAT2A_3
SUMF2_18		MAT2A_6
SYPL1_2		MLPH_6
TFPI_10		MMP24-AS1_5
TFPI_9		MMP7_2
TFRC_6		MORF4L2_4
TM4SF4_2		MYL6_3
TMCO1_2		NME4_5

TMCO1_3	NOP56_10
TMEM106B_4	NPTN_2
TMEM165_12	NT5DC2_6
TMEM9_5	NTAN1_4
TNNT1_5	OR51E1_2
TXNDC15_4	OSBPL9_15
UFL1_2	OSMR_2
VMP1_15	PCBP2_2
ZMPSTE24_1	PDAP1_2
ZNF358_1	PDCD5_8
	PFKP_4
	PGK1_2
	PLOD2_1
	PPP1R14B_1
	PPT1_5
	PROS1_1
	PSMC5_2
	PTPLAD1_2
	QPCT_6
	RAN_2
	RBM39_32
	RPLP0_16
	RPN1_3
	RPS24_1
	RPS2_4
	RPS2_6
	RTN4_10
	SAT1_6
	SEPT2_4
	SLC25A23_2
	SLC25A39_17
	SLC2A4RG_1
	SLC35A4_3
	SNF8_11
	SPP1_8
	SPTBN1_6
	SRM_1
	SRSF5_18
	TAF10_5
	TFPI2_1
	TFPI2_2
	TFPI_12

TM4SF1_3
TMCO1_5
TMED7_1
TMEM2_6
TMEM59_9
TPM2_3
TSR3_2
U2SURP_3
UCHL1_2
USF2_14
UXS1_6
VMP1_12
XBP1_2

Table S3. Topmost abundant cellular transcripts. The top 35 most abundant cellular transcripts are shown with their transcript ID (Ensembl) and Gene Symbol. The total amount of measured reads was recorded at each time point p.i.

Gene Symbol	Transcript_ID	Mock	6 h p.i.	12 h p.i.	24 h p.i.	48 h p.i.
MT-ND4_1	ENST00000361381	6247794	2853740	5086110	3492953	2729469
MT-CO1_1	ENST00000361624	3783091	1429703	3135777	2322469	2274467
MT-ND1_1	ENST00000361390	2709171	1030090	2052746	1455053	1268262
MT-ND2_1	ENST00000361453	2632634	1286921	1905244	1376563	1162716
MT-CO3_1	ENST00000362079	2359903	876498	1826921	1321628	1071133
ALDH1A1_2	ENST00000297785	1613839	987985	1530898	1163818	588438
FTH1_7	ENST00000526640	652381	243847	603476	416924	126432
FTL_1	ENST00000331825	473592	336371	456397	345480	163273
AKR1B10_4	ENST00000359579	468894	351460	518872	342488	146783
FTH1_10	ENST00000273550	362561	219428	291433	198129	108255
HSP90AA1_7	ENST00000216281	275709	172001	439806	353599	224562
YBX1_3	ENST00000321358	257240	131823	252684	187437	110877
AKR1C2_1	ENST00000380753	240398	136698	198116	163121	72093
RPL7_1	ENST00000352983	231994	118005	198785	162721	90750
FTH1_3	ENST00000534719	208654	166239	174550	132089	78161
RPL4_4	ENST00000307961	202204	107579	169998	141667	61741
NQO1_2	ENST00000379047	188594	137611	185722	138917	73311
FTH1_6	ENST00000532829	188097	126198	228284	135353	64559
AKR1C1_2	ENST00000380872	174789	97576	140911	118629	67087
RPL12_1	ENST00000361436	173224	111066	165750	128908	63271
ANXA2_18	ENST00000421017	163387	133822	150091	150557	37984
RPSA_9	ENST00000301821	151473	61395	106094	105900	53287
TRAM1_3	ENST00000262213	148467	104789	135438	93266	37472
RPLP1_2	ENST00000260379	132511	81784	116566	97361	52860
RPL23A_3	ENST00000422514	128191	-	124689	101409	52208
KRT8_1	ENST00000552551	127973	83720	126095	85336	-
RPL22_6	ENST00000234875	117534	52361	106623	81613	41268
PSAP_2	ENST00000394936	113331	-	93389	66862	54290
DCBLD2_4	ENST00000326840	110024	58437	127350	62553	-
S100A6_2	ENST00000368719	107117	59412	-	-	-
ACTB_10	ENST00000462494	104951	50185	115122	72080	50998
RPL37_2	ENST00000274242	104911	47567	105753	77556	39369
CA12_4	ENST00000178638	99505	-	85009	-	49915
HSPA8_7	ENST00000532091	98263	79707	111893	88139	-
SPP1_8	ENST00000395080	97083	62445	-	75002	-
TM4SF1_3	ENST00000305366	-	58813	-	-	-
RPLP0_10	ENST00000313104	-	58543	-	-	-
NAMPT_1	ENST00000222553	-	49532	79580	-	-
FADS1_16	ENST00000350997	-	-	81858	67268	39060
RPSA_1	ENST00000458478	-	-	-	65366	35135
CALR_1	ENST00000316448	-	-	-	-	45814
TM4SF20_2	ENST00000304568	-	-	-	-	38891
FN1_20	ENST00000492816	-	-	-	-	35248

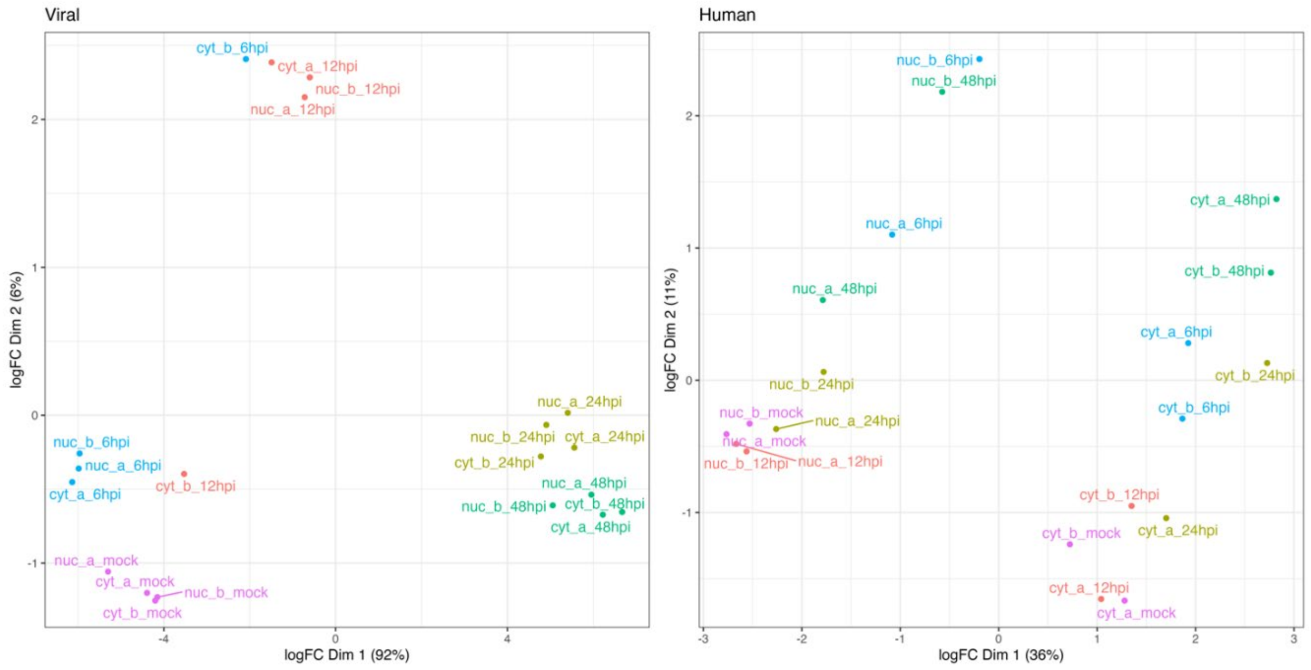

Figure S1. Principal component analyses. Shown are the correlation scatter plots of the PCA for nuclear and cytoplasmic samples at each time point of infection.

References

1. Zhao, H.; Chen, M.; Pettersson, U., A new look at adenovirus splicing. *Virology* **2014,** 456-457, 329-41.
2. Dix, I.; Leppard, K. N., Regulated splicing of adenovirus type 5 E4 transcripts and regulated cytoplasmic accumulation of E4 mRNA. *J Virol* **1993,** 67, (6), 3226-31.
